# Detection of Newcastle Disease Virus Minor Genetic Variants by Modified Single-Stranded Conformational Polymorphism Analysis

**DOI:** 10.1155/2014/632347

**Published:** 2014-04-10

**Authors:** Lukasz Rabalski, Krzysztof Smietanka, Zenon Minta, Boguslaw Szewczyk

**Affiliations:** ^1^Department of Recombinant Vaccines, Intercollegiate Faculty of Biotechnology, University of Gdansk, Medical University of Gdansk, Kladki 24, 80-822 Gdansk, Poland; ^2^The National Veterinary Research Institute, Aleja Partyzantow 57, 24-100 Pulawy, Poland

## Abstract

Newcastle disease and Avian Influenza are considered to be the most dangerous fowl diseases which may cause huge economic losses. Newcastle disease is caused by the enveloped, and single-stranded RNA virus (NDV, APMV-1; belonging to Paramyxoviridae family), which can be further divided into sixteen different genotypes grouped into five pathotypes according to their pathogenicity. It has been reported that low pathogenic virus can greatly increase its pathogenicity even during a single passage. Additionally, due to the widespread use of live vaccines, a mixture of two or more different viruses in one sample can be detected. Hence, there is a great need for establishment of fast, inexpensive, sensitive, and relatively simple diagnostic method for multistrain and quasispecies detection of NDV infection. In this paper we describe a diagnostic method based on RT-PCR followed by a modified version of single-stranded conformational polymorphism analysis using short DNA fragments of gene encoding viral F protein. The method allows for rapid diagnosis of genetic variant emerging from previously stable population which may prevent the spread of the pathogenic viral variant.

## 1. Introduction

Newcastle disease is an avian viral disease that endemically occurs in Asia, Africa, and Central and South America [1]. The etiological cause of the disease is an enveloped, nonsegmented, negative-sense, and single-stranded RNA virus belonging to* Avulavirus* genus of Paramyxoviridae family. This genus consists of twelve serotypes of Avian Paramyxoviruses [2]. Newcastle disease virus (NDV) is designated as APMV-1 [3]. It can be further divided into sixteen different genotypes [4, 5].

NDV genome contains 15186, 15192, or 15198 nucleotides depending on genotype and encodes six proteins in the following order: NP (nucleocapsid protein), P (phosphate polymerase cofactor protein), M (membrane associated matrix protein), F (fusion surface glycoprotein), HN (hemagglutinin-neuraminidase surface glycoprotein), and L (large RNA dependent RNA-polymerase) [6–8]. Paramyxovirus particles usually enter the host organism via the respiratory or gastrointestinal epithelial cells. F protein is activated by HN protein which promotes junctions and fusion of viral and host membranes [9]. Matrix protein dissolves and releases helical nucleocapsid into the cytoplasm of the infected cell [10]. After transcription and translation, newly formed progeny virions bud out of the cell [11].

Newcastle disease is widely spread throughout the globe and is considered to be one of the two most dangerous poultry diseases (second is the avian influenza) that cause huge economic losses in poultry production. The loss of US$161 million by the US Government in 2002 after the outbreak of Newcastle disease in California is one of the most recent cases of the disease.

The name of the disease is derived from the British city “Newcastle-upon-Tyne,” near which it was diagnosed in 1926 for the first time. Almost simultaneously, in the same year, the disease was discovered on Java Island in Indonesia [12]. It was experimentally proven that NDV infects at least 236 avian species [13]. Human infections are rare and usually exhibit as eye conjunctivitis; several infections in pigs have been reported [14, 15]. Naturally selected or laboratory adapted strains of the NDV are thoroughly studied, some of them found application as new oncolytic virotherapeutics that can be used to treat human cancers like melanoma [15, 16].

The main reservoirs of the APMV-1 are waterfowl and migratory birds [17, 18]. During infection with low pathogenic strains often they do not exhibit clinical signs, which allows for the “unnoticed” spread of the virus. The highest mortality (even higher than 90% in 2–6 dpi) among infected birds is observed in domestic fowl (*Gallus gallus domesticus*) [1]. Virulence of a strain depends mainly on the short amino acid sequence of the precursor protein responsible for fusion of membranes [19]. Highly pathogenic NDV usually contains the following sequence: C-^112^R/K-R-Q/K/R-R/K-R-F^117^-N, while low pathogenic has C-^112^G/E-K/R-Q-G/E-R-L^117^-N. These motifs are recognized by different cellular proteases which limit the ability for virus propagation only to specific host tissue [20, 21]. World Organisation for Animal Health distinguishes five pathotypes of Newcastle disease virus: viscerotropic and neurotropic velogenic (highest mortality rate, Intracerebral Pathogenicity Index > 1,5), mesogenic (low mortality but moderate signs from respiratory system, ICPI 1,5–0,7), lentogenic (mild respiratory infection with no mortality, life-vaccine strains, ICPI < 0,7), and asymptomatic (no signs or subclinical enteric infections) [22].

Although vaccines based on low pathogenic variants of NDV protect against clinical signs and mortality, they do not prevent infection with highly pathogenic strains, which may be the cause for uncontrolled spread of the virus [23]. This phenomenon may be intensified if chickens are vaccinated at the same time against other viruses (e.g., against infectious bursal disease virus) or if the dose of the vaccine is too small and/or poorly administered [24–26].

Molecular diagnostic methods that employ different variations of RT-PCR and real-time PCR techniques allow for sensitive detection and pathotyping of APMV-1 [27–37]. All techniques based on endonuclease cleavage or probe/primer hybridization to a specific site are very sensitive to mismatches that often produce false-negative results [37, 38]. Methods that use high resolution or standard melting curve analysis are unable to detect multistrain or quasispecies infection without very expensive, time and resource consuming preanalysis [39]. These deficiencies are the most important after the discovery that highly virulent viruses may arise from progenitor viruses of low virulence, as it was shown during Australian 1998–2002 outbreak [40, 41]. Even a single passage in chicken's brain can change virus pathotype to velogenic [42, 43].

**Figure 1:**
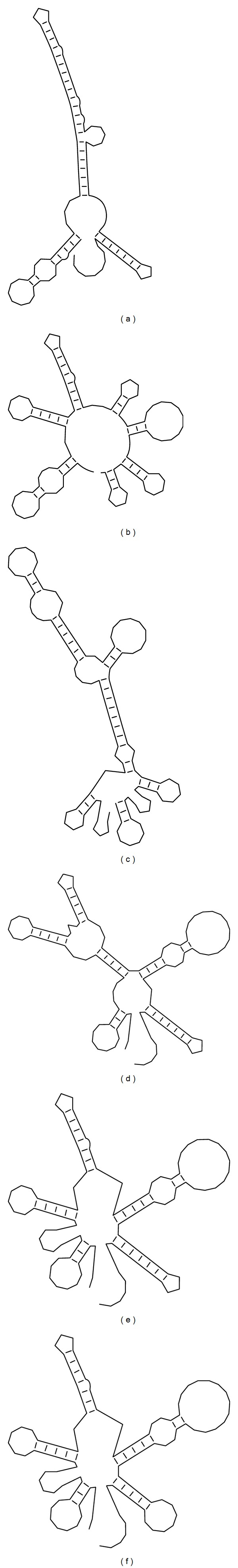
Possible ssDNA conformers of 123 nt fusion protein fragments of La Sota (a), Roakin (b), Italy (c), lentogenic 98-1154 (d), mesogenic 99-0868-2 (e), and velogenic 99-0655 (f) NDV strains. Structures were drawn in Geneious R6 PRO software using ssDNA energy model at 20°C.

One of the methods which may help in differentiation as well as in quasi- and multistrain NDV detection is one-step RT-PCR followed by single-stranded conformational polymorphism. Single-stranded conformational polymorphism (SSCP) analysis was first described by Orita et al. [44] who demonstrated that single-stranded DNA fragments with a single base difference can be potentially distinguished on the basis of difference in electrophoretic mobility under native conditions. During the gel run, single-stranded fragments adopt secondary structure depending on the sequence and conditions. Exemplary ssDNA secondary structures are presented in Figure 1. Usually more than one electrophoretic band of ssDNA is observed for a particular DNA fragment due to the fact that often more than one conformer is thermodynamically favorable in experimental conditions. Physicochemical conditions, the most important being ionic strength, pH, and temperature, play an important role in the formation of particular conformers [45–47]. In the modification of this technique named multitemperature single-stranded conformational polymorphism (MSSCP), the electrophoretic gel is run at stringently controlled temperatures which are changed stepwise during the run [48]. SSCP analysis has been recently applied to the identification of genetic variation in several groups of viruses [49–51]. One of the most useful applications of this technique has been in the identification of quasispecies within the population of viruses making it an effective tool in molecular epidemiology studies [52, 53].

The purpose of this study was to develop a method for inexpensive, quick, and sensitive identification of NDV variants with a point mutation in gene F cleavage site that can lead to increase of the pathogenicity. Ideally, the method should allow for detection of minor quasispecies at relatively low cost in routine diagnostic laboratories.

## 2. Materials and Methods

### 2.1. APMV-1 Strains

Full size or fragments of genomes from three reference strains (vaccine La Sota, vaccine Roakin, and Italy) and three Australian strains (isolated during 1998–2002 outbreak in New South Wales) were tested in this study and are listed in Table 1.

### 2.2. Template Preparation, RT-PCR Conditions

nR—allantoic fluid from SPF embryonated flock eggs (Lohman, Germany), previously inoculated with La Sota or Roakin NDV strains, was harvested and subjected to RNA extraction using RNeasy Mini Kit according to the manufacturer protocol (Qiagen, USA). RNA from oral and cloacal swabs from chickens infected with La Sota or Roakin NDV strains was purified with the same procedure.

nD—one set of universal (for all Class II APMV-1) degenerate PCR primers for amplification of 123 nt fusion glycoprotein gene fragment was designed. The DNA sequences for this set of primers were as follows: NDV-F (position 4790–4809 in La Sota genome): 5′-GCATACAACAGRACAYTGAC-3′ and NDV-R (position 4893–4912 in La Sota genome): 5′-GCCDATAATGGCRCCTATAA-3′. Synthesis of the first strand and amplification of the DNA were carried out during one-step RT-PCR (AccessQuick RT-PCR System, Promega, USA) procedure. The reaction mixture contained 1 *μ*L template RNA, 12.5 *μ*L AccessQuick Master Mix, 0.5 *μ*L 10 *μ*M NDV-F primer, 0.5 *μ*L 10 *μ*M NDV-R primer, 0.5 *μ*L (2.5 u) AMV Reverse Transcriptase, and 10.5 *μ*L Nuclease-Free Water. The reaction conditions were as follows: 1x [45°C 40 min., 95°C 2 min.], 30x [95°C 30 sec., 43°C 30 sec., 70°C 15 sec.], and 1x [70°C 2 min]. PCR products were electrophoresed in 2% agarose gels using 1x TAE buffer (40 mM TRIS, 20 mM sodium acetate, and 1 mM EDTA adjusted to pH 7.2 with glacial acetic acid) with ethidium bromide and purified (Gel-Out Kit, A&A Biotechnology, Poland) and cloned into the pJet 1.2 vector (CloneJET PCR Cloning Kit, Thermo Scientific, USA) in accordance with the manufacturers protocols. The recombinant plasmids were used to transform the TOP10 strain of* E. coli* competent cells (Life Technologies, USA). Colonies were isolated and they were grown in liquid medium for purification of recombinant plasmids on silica gel columns (Plasmid Mini, A&A Biotechnology, Poland). After restriction analysis, the recombinant plasmids containing the correct inserts were sent for sequencing to Genomed, Poland. Sequences were aligned using the Geneious R6 PRO software created by Biomatters, available from http://www.geneious.com/.

aD—three 145 bp DNA oligonucleotides with sequences exactly the same as those in Australian strains of NDV (position 4790–4934 in La Sota genome) presented in Table 1 were synthetized in Genomed, Poland. These fragments were subjected to cloning, restriction analyses, and sequencing as described above.

aR—all purified and checked for correct sequence plasmids were used as template for transcription reaction by TranscriptAid T7 High Yield Transcription Kit (Thermo Scientific, USA) as instructed by the manufacturer. RNA was checked for lack of DNA contamination by RT-PCR as described above without addition of AMV Reverse Transcriptase.

### 2.3. MSSCP Analyses

Templates nR and aR were subjected to RT-PCR and plasmids (nD and aD) to PCR (no initial 45°C 40 min. step or AMV Reverse Transcriptase). For MSSCP analysis, 3 *μ*L of DNA was added to 10 *μ*L of denaturing buffer (0.1 M NaOH and 10 mM EDTA) and incubated at 98°C for 5 min. The samples were immediately cooled on ice and, prior to loading on the gel, 3 *μ*L of dye solution (0.1% bromophenol blue, 0.1% xylene cyanol in formamide) was added. The mixture was immediately loaded onto 13.5% or 11% native polyacrylamide gel (and with addition of 3% glycerol for 13.5% gel). Electrophoresis was carried out in 0.75x TBE (45 mM Tris, 45 mM boric acid, 1 mM EDTA, and pH 8.0) in DNA Pointer System (BioVectis, Poland) at three or five different temperatures. Before transferring samples onto the gel, a 100Vxh 9°C preelectrophoresis was performed. The MSSCP electrophoresis conditions are shown in Table 2. After electrophoresis, gels were stained using the Silver Stain Kit from BioVectis.

## 3. Results

### 3.1. Reverse Transcription PCR and Validation of Templates

During the experiments, different types of templates for polymerase amplification reactions were tested (Table 1). For validation of these templates, one-step RT-PCR and PCR were performed. Full genome (nR) or fusion protein fragment (aR, nD, and aD) templates were subjected to amplification with one set of universal degenerate primers (NDF-F and NDF-R). For RT-PCR only RNA (both full genomic and F protein fragment transcribed from a plasmid) and for PCR only DNA (a plasmid with F protein fragment) were used. No differences were observed in the resulting products.

**Figure 2:**
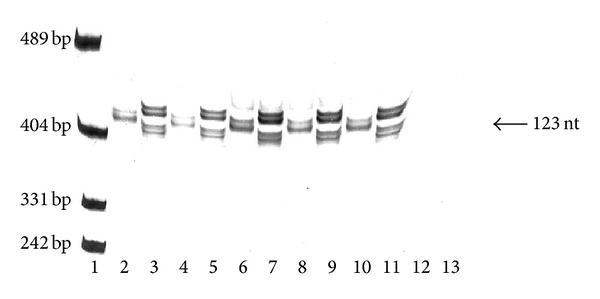
Multitemperature single-stranded conformational polymorphism (MSSCP) electrophoresis of 123 nt fusion protein fragments of two vaccine Newcastle disease virus strains obtained from RT-PCR (lanes 2–5 and 8–11) and PCR (lanes 6 and 7). Types of templates (nR, aR, and nD) were described in Section 2. Lanes: 1. Double-stranded pUC19 DNA/MspI Marker, 23 (Thermo Scientific, USA), 2. Oral swab nR La Sota, 3. Oral swab nR Roakin, 4. Cloacal swab nR La Sota, 5. Cloacal swab nR Roakin, 6. Plasmid nD La Sota, 7. Plasmid nD Roakin, 8. Allantoic fluid nR La Sota, 9. Allantoic fluid nR Roakin, 10. Transcribed RNA aR La Sota, 11. Transcribed RNA aR Roakin, 12. RT-PCR negative control, 13. PCR negative control. MSSCP was performed as described in Table 2 without the first two temperatures steps.

Until now no data have been presented whether the preamplification procedures (like using only small fragment of transcribed RNA instead of full genomic RNA for RT-PCR) affect secondary ssDNA structures and their quantity, which subsequently may alter MSSCP band pattern. To check this, MSSCP analysis for RT-PCR products derived from different templates was conducted. The result is shown in Figure 2 which indicates clearly that there is no differences in ssDNA patterns irrespective of its preparation. This validates the use of synthetic DNA representing NDV strain or genetic variant, instead of viral genome for establishing MSSCP bands pattern.

### 3.2. Detection and Differentiation of “Shedding” and Quasispecies Infections

**Figure 3:**
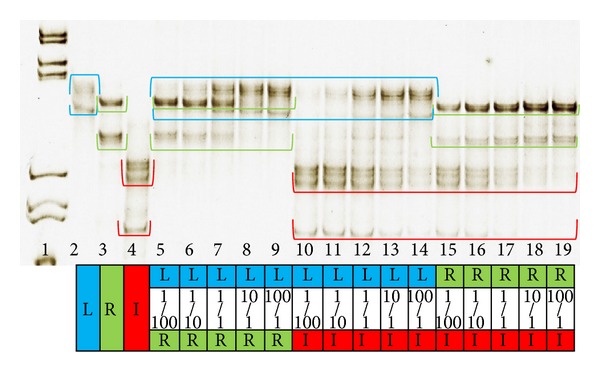
MSSCP electrophoresis of 123 nt NDV fusion protein fragments RT-PCR and PCR products. Lanes 2–4 are RT-PCR products (genomic RNA—nR template; L—La Sota strain, R—Roakin strain, and I—Italy strain) where lanes 5–19 are PCR products (plasmids with cloned fusion protein gen fragment—nD and aD templates). The PCR strains sample composition, plasmid ratio, and color code explanation can be found in the table below the figure. MSSCP was performed in 11% polyacrylamide gel without any additives as described in Table 2.

**Figure 4:**
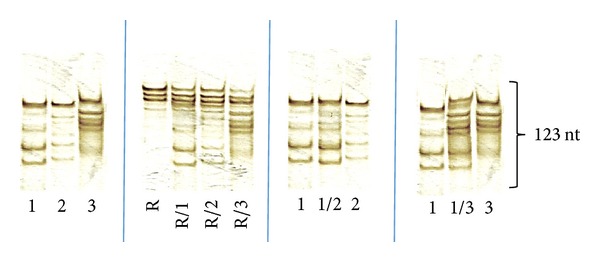
MSSCP electrophoresis of 123 nt NDV fusion protein fragments RT-PCR products (nR and aR templates). Labeling: 1. Australian lentogenic strain 98-1154, 2. Australian mesogenic strain 99-0868-2, 3. Australian velogenic strain 99-0655, R. Vaccine mesogenic Roakin strain. MSSCP was performed in 13,5% polyacrylamide gel with addition of 3% glycerol as described in Table 2.

To test the ability to detect two different NDV variants in a single sample, a series of mixed template PCRs was prepared. After reaction, MSSCP analysis was performed and ssDNA band patterns were visualized as presented in Figures 3 and 4. The presence of all Australian genetic variants was clearly detected in combinations with each other and with vaccine Roakin strain.

## 4. Discussion

Low pathogenic strains of NDV in feces of infected birds can remain active for more than two months, and the virus can persist for over two years in bird population [54–56]. This has not raised much concern until the last decade of 20th century when it was found that a low pathogenic virus can transform into highly pathogenic with only four point mutations. Such spontaneous change was documented for the first time for Irish velogenic isolates in 1990 [57, 58]. At that time, Australia was considered as a continent free from highly pathogenic strains of APMV-1 [59]. However, ten years later, a similar change in virulence was observed during the outbreak that took place in 1998–2002 in the New South Wales, Australia. Two point mutations in the cleavage site of the F glycoprotein resulted in a complete change of pathotype and threatened poultry farms on the continent. It has been found that the viral population circulating in the environment consisted of a number of intermediate forms, called quasispecies [41, 60]. The idea of constantly changing (mutating) viruses that generate different properties (e.g., virulence) in subpopulations was previously confirmed for many viruses not related to NDV [61–63]. This specific feature is most pronounced in RNA viruses because of lack of proofreading properties of RNA polymerase to efficiently repair genomes that undergo replication [64, 65].

The Australian case gave rise to the discussion whether it is possible to detect such events in advance. To cope with the problem we need to establish a method that will meet some basic requirements. This method must be able to detect numerous genetically altered variants in the pool of progenitor viruses as well as it must be able to distinguish between two sequences with only single nucleotide mismatch. Additional advantage of such method should be low cost and simplicity.

The combination of Reverse Transcription real-time, digital PCR with Sanger sequencing or applications of Next Generation Sequencing and oligonucleotide microarrays can give great results, but they are expensive and highly demanding for trained technician and elaborate equipment [39, 66, 67]. As an alternative, many protocols, based on techniques like restriction endonuclease analyses, peptide nucleic acid with gold nanoparticles assay, phage-capturing dot blot, in situ hybridization, RT-PCR, and probe-based real-time RT-PCR, were proposed [68–72]. Their main limitation is the need to react with a specific nucleotide sequence responsible for virulence. Single nucleotide mismatches that are responsible for acquiring virulence may not be detected and can lead to false diagnostic results [37, 38].

During our study, we developed a method, based on RT-PCR followed by MSSCP analyses, that meets the requirements described in the second paragraph. In our opinion, fast diagnostics of a new genetic variant that appears in previously stable population could alert veterinary services and lead, after confirmation, to preventive actions for disease eradication. MSSCP can differentiate between lentogenic vaccine, mesogenic vaccine, circulating velogenic, lentogenic natural occurring, and their highly pathogenic mutated NDV variants. This can be achieved by comparison of unknown pattern of silver stained bands to previously established database of known isolates. Even without direct information about pathotype of the unknown sample, it is easy to distinguish potentially unwanted strain of NDV. The technique described above does not require highly sophisticated and expensive tools and is easy to perform by laboratory technical personnel. Additionally it is relatively inexpensive and takes about 5 minutes per sample from the end of one-step RT-PCR until visualization. Main disadvantage (that has no significance for a technician performing the assay) of MSSCP is a lengthy and arduous optimization of gel run conditions like selection of optimum conditions (temperature, voltage, and time) and gel percentage of polyacrylamide and other additives.

## 5. Conclusions

Many methods have been developed that allow for detecting different pathotypes of Newcastle disease virus. One of the most popular and validated molecular techniques is real-time PCR. The disadvantage of this method is difficulty in detection of multistrain infections and minute changes in sequence that can alter virulence. In this study, we described a method based on a modification of single-stranded conformational polymorphism at specially programmed temperatures (MSSCP) that can be easily applied for NDV screening. We also showed that to prepare a library of MSSCP patterns it is not necessary to use isolated genomic material but only short synthetized nucleotides. In other words, it is possible to create a database of ssDNA band patterns for all-known NDV F gene sequences (e.g., available in NCBI) without need to isolate their genetic material but by synthetizing dsDNA analogs and reverse transcription to RNA. They can be used as a template for RT-PCR and produce exactly the same MSSCP ssDNA band patterns (Figure 2).

## Figures and Tables

**Table 1:** List of NDV strains with different types of templates for PCR or RT-PCR and highlighted point mutations affecting their virulence.

Strain	Templates*	GenBank acc. number	Nucleotide motif**	ICPI	Pathotype
La Sota	nR, aR, nD	AF077761	GGGAGACAGGGGCGCCTT	0.4 [1]	Lentogenic
Roakin	nR, aR, nD	AY289000	AGGAGACAGAAACGCTTT	1.45 [1]	Mesogenic
Italy	nR, aR, nD	AY562989	AGGAGGAAGAAACGCTTT	1.55 [73]	Velogenic
98-1154	aR, aD	AY935491	AGGAGACAG**G**GGCGT**C**TT	0.47 [60]	Lentogenic
99-0868-2	aR, aD	AY935496	AGGAGACAG**A**GGCGT**C**TT	1.38 [60]	Mesogenic
99-0655	aR, aD	AY935494	AGGAGACAG**A**GGCGT**T**TT	1.61 [60]	Velogenic

*nR: genomic RNA isolated from live-virus vaccines, aR: RNA obtained by transcription of cloned fusion protein gene fragment from a plasmid, nD: plasmids with cloned cDNA from RT-PCR of genomic RNA, and aD: plasmids with cloned synthetized fusion protein gene fragment.

**nucleotide sequence for F_0_ cleavage site (position 4877–4894 in La Sota genome).

**Table 2:** Conditions of MSSCP electrophoresis.

Step	Vxh	Temp. (°C)
1	600	9
2	550	13
3	500	17
4	450	20
5	400	23
